# Superior vena cava obstruction presenting with epistaxis, haemoptysis and gastro-intestinal haemorrhage in two men receiving haemodialysis with central venous catheters: two case reports

**DOI:** 10.1186/1752-1947-3-6180

**Published:** 2009-05-27

**Authors:** Seerapani Gopaluni, Paul Warwicker

**Affiliations:** 1 Renal Unit, Lister Hospital, Stevenage, SG1 4AB, UK

## Abstract

**Introduction:**

Superior vena cava (SVC) obstruction secondary to central venous catheterization is an increasingly recognized complication.

**Case presentation:**

We present two cases of superior vena cava obstruction secondary to indwelling central venous catheters used for haemodialysis access. One of the patients developed the unusual complications of torrential epistaxis and haemoptysis, which has been reported only once so far in the literature. The other patient developed melaena secondary to downhill oesophageal varices. We briefly discuss the pathophysiology, symptoms and signs, investigations and management of superior vena cava obstruction and thrombosis.

**Conclusion:**

Increasing use of central venous access for haemodialysis will increase the incidence of central venous stenosis, thrombosis and exhaustion. Superior vena cava obstruction is likely to be an increasingly recognised complication of vascular access in the future.

## Introduction

Superior vena cava (SVC) obstruction and thrombosis caused by indwelling venous catheters is a growing problem, and is associated with an appreciable morbidity and mortality. We present two cases of SVC obstruction secondary to multiple central venous catheterizations. In the first case this was complicated by haemoptysis and torrential epistaxis. In the second this was complicated by 'downhill' oesophageal varices, and gastro-intestinal bleeding, compounded by anti-coagulation.

## Case presentations

### Case 1

A 53-year-old man on haemodialysis presented with a one-week history of worsening shortness of breath, facial and arm swelling. He was receiving dialysis through his right jugular vein via a tunnelled catheter. He had previously undergone multiple vascular access procedures for haemodialysis. He was on long-term anticoagulation for repeated thrombotic complications. A week prior to his admission his warfarin had been temporarily stopped to facilitate peritoneal dialysis catheter insertion.

**Figure 1:**
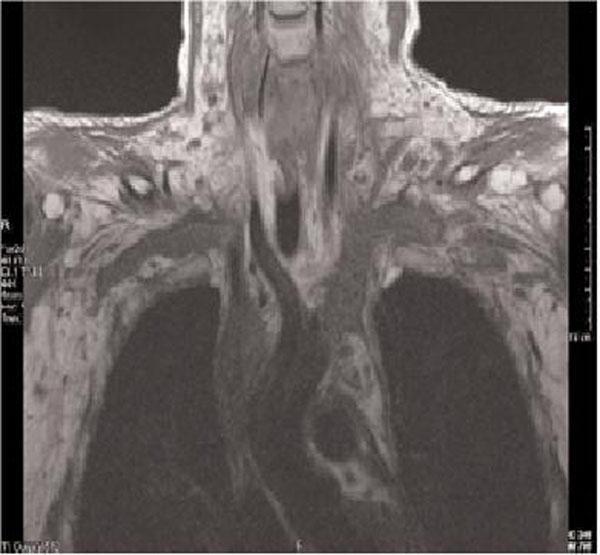
**Magnetic resonance venography of central veins of patient number one**. There is loss of normal flow void with extensive echogenic material in the SVC and both brachiocephalic veins extending into the subclavians.

A clinical diagnosis of SVC obstruction was made and warfarin was restarted. This was confirmed on magnetic resonance venous imaging, which showed extensive thrombosis in the superior vena cava extending into both brachiocephalic veins (Figure 1). Shortly afterwards he developed significant haemoptysis and persistent epistaxis, eventually requiring tracheal intubation and respiratory support. The bleeding persisted despite reversing the anticoagulation and anterior and posterior nasal packing, bilateral spheno-palatine artery ligation and cauterization of bleeding venous sites. He subsequently died from complications.

### Case 2

A 75-year-old man who presented with marked swelling of his face and arms, shortness of breath on exertion and lethargy. Two weeks earlier he had the fifth re-insertion of a tunnelled right internal jugular haemodialysis catheter. He also had a history of failed vascular access procedures, including arterio-venous fistulas and synthetic grafts.

**Figure 2:**
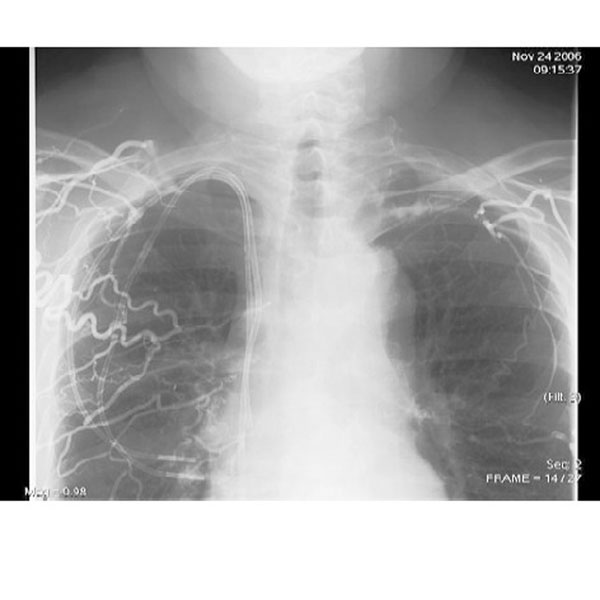
**Bilateral simultaneous arm venography (Patient 2) demonstrating multiple collateral veins, but no opacification of the central veins**.

**Figure 3:**
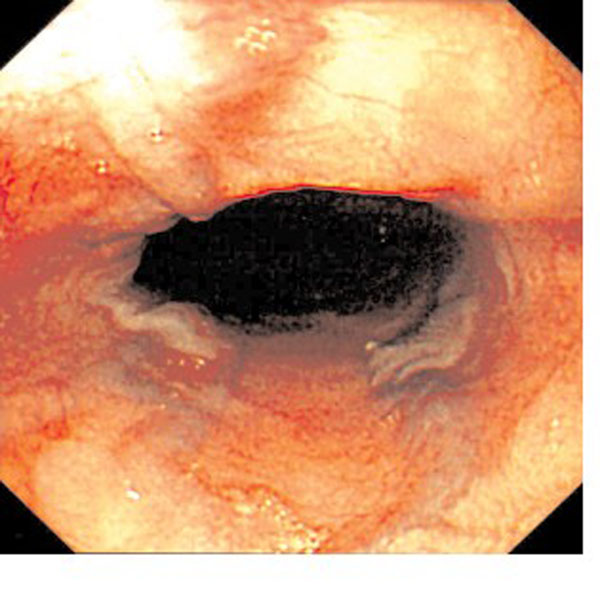
**Endoscopic appearance of distended veins in the proximal oesophagus (downhill varices) in patient number two**.

Again a clinical diagnosis of SVC obstruction was made and warfarin was started. An attempted superior vena cavagram was unsuccessful, despite injecting dye into both arm veins - which demonstrated multiple collateral veins, but no opacification of the central veins at all (Figure 2). However, subsequent magnetic resonance venography demonstrated a SVC stenosis, occluded by a clot surrounding his tunnelled venous catheter. A decision was made to anticoagulate him for a period of four to six weeks before attempting to withdraw the catheter, and in the interim to start dialysis via a tunnelled femoral catheter. Two weeks later he presented with melaena. His haemoglobin had fallen from 11.6 to 6.3 g per dl. He was transfused and his anti-coagulation was reversed. Emergency endoscopy revealed enlarged and bleeding oesophageal 'downhill' varices (Figure 3). 'Downhill' oesophageal varices, in the upper third of the oesophagus, are less common than classical 'uphill' varices, caused by portal hypertension and found in the lower third [1,2]. His jugular catheter was removed without any complications, under fluoroscopic screening and subsequently the bleeding settled. Following the catheter removal the patient was well and the swelling improved.

## Discussion

Worldwide, a combination of under-provision of vascular access surgery, late referral and co-morbidity cause increasing numbers of our patients to utilize tunnelled venous catheters in the medium to long term. One consequence of this will be the increasing incidence of central venous stenosis, thrombosis and exhaustion. SVC obstruction is likely to be an increasingly recognised complication of vascular access in the future.

The pathophysiology is thought to be secondary to early intimal injury associated with focal endothelial denudation occurring with short-term central venous catheters [3], and related to the position of the tip of the catheter, the site of insertion, the material, and predisposition to thrombosis [4]. With long-term catheter use, there is vein wall thickening, increased smooth muscle cells and focal catheter attachments to the vein wall with thrombus and collagen.

## Conclusion

Clinicians should be aware of the clinical consequences of SVC obstruction, which include facial and arm swelling, dyspnoea, headache, dysphagia, cough, visual disturbance but also, less commonly, hoarseness, epistaxis, syncope, tongue swelling, upper gastro-intestinal bleeding and haemoptysis. Common signs include face and neck swelling, upper extremity swelling and dilated chest collaterals.

Angiography, indirect evidence from ultrasonography [4] and MRI are used to establish the diagnosis of SVC obstruction.

Management needs to be individualized. In the first few days of SVC thrombosis, removal of catheter, chemical or mechanical thrombolysis of the clot [5] and/or venoplasty and stenting [6] has been reported to resolve the symptoms. Thereafter, the chance of success is diminished, and formal anticoagulation may run the risk of exacerbating bleeding from engorged collateral veins.

In both of our cases the risk of doing nothing (thrombolysis and anti-coagulation being contraindicated by the life threatening bleeding that characterised both patients) was weighed against the potential benefit of reducing the pressure dynamics of the upper body central veins. Although we were all aware of the risk - it is notable that the patient whose catheter was removed, survived. The risk arising from thromboembolism, of early removal of catheters, has been highlighted [4].

## Abbreviations

SVC: superior vena cava; MRI: magnetic resonance imaging.

## Consent

Written informed consent was obtained from the patients (or next of kin) for publication of this case report and accompanying images. A copy of the written consent is available for review by the Editor-in-Chief of this journal.

## Competing interests

The authors declare that they have no competing interests.

## Authors' contributions

SG did the literature search and contributed to writing the majority of the case reports. PW managed the patients, edited the case reports and obtained the figures. Both authors read and approved the final manuscript.
